# Polyphyly of the hawk genera *Leucopternis *and *Buteogallus *(Aves, Accipitridae): multiple habitat shifts during the Neotropical buteonine diversification

**DOI:** 10.1186/1471-2148-6-10

**Published:** 2006-02-07

**Authors:** Fabio S Raposo do Amaral, Matthew J Miller, Luís Fábio Silveira, Eldredge Bermingham, Anita Wajntal

**Affiliations:** 1 Departamento de Genética e Biologia Evolutiva, Universidade de São Paulo, São Paulo. Rua do Matão, 277, Cidade Universitária, São Paulo, SP, CEP 05508-900, Brasil; 2 Smithsonian Tropical Research Institute, Apartado 2072, Balboa, Panamá; 3 Departamento de Zoologia, Universidade de São Paulo, São Paulo. Rua do Matão, Travessa 14, n° 321, Cidade Universitária, São Paulo, SP, CEP 05508-900, Brasil

## Abstract

**Background:**

The family Accipitridae (hawks, eagles and Old World vultures) represents a large radiation of predatory birds with an almost global distribution, although most species of this family occur in the Neotropics. Despite great morphological and ecological diversity, the evolutionary relationships in the family have been poorly explored at all taxonomic levels. Using sequences from four mitochondrial genes (12S, ATP8, ATP6, and ND6), we reconstructed the phylogeny of the Neotropical forest hawk genus *Leucopternis *and most of the allied genera of Neotropical buteonines. Our goals were to infer the evolutionary relationships among species of *Leucopternis*, estimate their relationships to other buteonine genera, evaluate the phylogenetic significance of the white and black plumage patterns common to most *Leucopternis *species, and assess general patterns of diversification of the group with respect to species' affiliations with Neotropical regions and habitats.

**Results:**

Our molecular phylogeny for the genus *Leucopternis *and its allies disagrees sharply with traditional taxonomic arrangements for the group, and we present new hypotheses of relationships for a number of species. The mtDNA phylogenetic trees derived from analysis of the combined data posit a polyphyletic relationship among species of *Leucopternis*, *Buteogallus *and *Buteo*. Three highly supported clades containing *Leucopternis *species were recovered in our phylogenetic reconstructions. The first clade consisted of the sister pairs *L. lacernulatus *and *Buteogallus meridionalis*, and *Buteogallus urubitinga *and *Harpyhaliaetus coronatus*, in addition to *L. schistaceus *and *L. plumbeus*. The second clade included the sister pair *Leucopternis albicollis *and *L. occidentalis *as well as *L. polionotus*. The third lineage comprised the sister pair *L. melanops *and *L. kuhli*, in addition to *L. semiplumbeus *and *Buteo buteo*. According to our results, the white and black plumage patterns have evolved at least twice in the group. Furthermore, species found to the east and west of the Andes (*cis-*Andean and *trans-*Andean, respectively) are not reciprocally monophyletic, nor are forest and non-forest species.

**Conclusion:**

The polyphyly of *Leucopternis*, *Buteogallus *and *Buteo *establishes a lack of concordance of current Accipitridae taxonomy with the mtDNA phylogeny for the group, and points to the need for further phylogenetic analysis at all taxonomic levels in the family as also suggested by other recent analyses. Habitat shifts, as well as *cis- *and *trans*-Andean disjunctions, took place more than once during buteonine diversification in the Neotropical region. Overemphasis of the black and white plumage patterns has led to questionable conclusions regarding the relationships of *Leucopternis *species, and suggests more generally that plumage characters should be used with considerable caution in the taxonomic evaluation of the Accipitridae.

## Background

The family Accipitridae comprises approximately 237 species of predatory birds distributed worldwide except Antarctica [1], with diversity concentrated in the Neotropics [1,2]. Despite numerous taxonomic revisions (e.g., [3,4]), the evolutionary history of the family has not been sufficiently explored using methods of phylogenetic inference, and current classifications are mainly based on plumage and ecological resemblance between taxa [5]. Current taxonomy is still highly provisional at all taxonomic levels [1,6], and does not appear to reflect phylogenetic relationships in several cases [7,8], thus retarding biogeographic analysis, morphological trait mapping and the general understanding of the evolutionary history of the Accipitridae.

The Accipitridae morphological diversity has been traditionally represented in sub-groups of similar or supposedly closely related species, such as "kites", "harriers", "booted eagles" and "buteonines" [1]. The buteonine hawks are represented by the large cosmopolitan genus *Buteo *and several related genera, called "sub-buteonines" by Amadon [4], which includes the predominantly Neotropical genera *Buteogallus, Parabuteo, Asturina, Leucopternis, Busarellus, Geranoaetus, Geranospiza *and *Harpyhaliaetus*. Two old world genera, *Kaupifalco *and *Butastur*, were formerly included as part of the "sub-buteonines" group, but were subsequently removed from this division [9]. Some authors consider the buteonine as a sub-family (Buteonineae, *e.g*. Friedman [10], Grossman and Hamlet [11]), but formal sub-familial division of Accipitridae has been a contentious issue due to a lack of knowledge of the evolutionary history of the family (see [9,12]).

Evolutionary biologists have long sought to understand the processes responsible for the generation of the high species richness found in the Neotropics, and several models of biotic diversification have been invoked to explain such patterns, for example forest refuges resulting from climatic fluctuations [13-15], rivers as barriers to gene flow [16], river dynamics [17], sea level oscillations [18-20], geotectonic vicariance [21] and ecological factors [22] (see Moritz *et al*. [23] for a revision). However, these models have only rarely been tested with organisms capable of long-distance dispersal (e.g., [24,25]), such as hawks and eagles capable of soaring and gliding flight. Numerous flocks of migrant hawk species as *Buteo platypterus *and *Buteo swainsoni*, for example, cross the Andes as part of their yearly migrations [26], and call into question the degree to which the geographical barriers to gene flow identified in many models of Neotropical diversification have been important in Accipitridae speciation.

The genus *Leucopternis *is a morphologically heterogeneous group of 10 buteonine species distributed in forested habitats from southern Mexico to Paraguay and Uruguay [1], and offers an opportunity to explore the diversification of an Accipitridae group distributed throughout the Neotropical region. Species in the genus vary from the small *L. semiplumbeus *(250 g) to the large *L. princeps *(1 kg) [1], and are hawks with broad wings and medium to short tails. Two species, *L. schistaceus *and *L. plumbeus*, are entirely dark slate; however, most *Leucopternis *have primarily white plumage and vary in the amount of black, grey or slate black on the back, wings and/or head. Those which we here refer to as "black-and-white" *Leucopternis *species are *L. albicollis*, *L. polionotus, L. occidentalis, L. lacernulatus, L. melanops, L. kuhli*, *L. semiplumbeus*, and *L. princeps *[1,6]. While *Leucopternis *are found exclusively in forest habitats, other Neotropical buteonine species occur in a variety of habitats, such as mangroves (*Buteogallus aequinoctialis*), savannahs (*Harpyhaliaetus coronatus, Buteogallus meridionalis*) and wetlands (*Busarellus nigricollis*) [1,6], which makes this group suitable to analysis of the evolutionary relationships of forest and non-forest species.

The buteonine phylogeny has been partially explored recently using morphological and molecular data [7,27,28], but Neotropical species have not been well represented. Incomplete taxon sampling notwithstanding, these analyses have called into question the monophyly of *Leucopternis*, *Buteo *and *Buteogallus *[7,28]. The present work constitutes an effort to clarify the relationships among all *Leucopternis *species, and their position relative to other Neotropical buteonine genera. We address the following questions: (1) Is *Leucopternis *as currently recognized monophyletic? (2) What are the relationships among species of *Leucopternis *to other genera of buteonine hawks? (3) Is the black and white plumage pattern a synapomorphic trait uniting the majority of species in the genus *Leucopternis*? (4) Are phylogenetic relationships among Neotropical buteonines predicted by biogeography or habitat?

## Results

### Datasets, molecular variation

Our final alignment of the total dataset (12S, ATP8 and 6 and ND6) without gaps totalled 2179 base pairs, with 651 variable and 505 parsimony informative sites. Uncorrected distances ranged from 0 to 7.1% for 12S (without gaps), 0 to 21.4% for ATP8, 0 to 11.9% for ATP6, and 0 to 13.9% for ND6. Deviations from linearity were found in third position plots of ATP8, ATP6 and ND6. We did not detect significant departures from homogeneity of base frequencies across taxa in any dataset (P > 0.05, data not shown). We are confident of mitochondrial origin of our sequences because: (1) most of our samples were represented by mitochondrial-rich tissues (feathers, muscle or liver); (2) most samples (comprising 12 of 20 species) had all regions sequenced using independent fragments amplified with different primer sets (with sequence overlap ranging from 67 to more than 400bp, in highly variable regions), and sequences were identical; (3) sequences were easily aligned to published sequences of other Accipitridae species; (4) electropherograms were carefully checked for double peaks; (5) coding regions did not show unexpected stop codons; and 6) gene specific phylogenetic analyses revealed similar relationships to those inferred from the combined data, indicating that a mitochondrial translocation to the nucleus would have to have been more than eight kilobases in length.

A single nucleotide site in the 12S sequence of the muscle sample LGEMA F39 (*L. lacernulatus*) presented a strong "C" peak with a lower "A" peak at the position 593, and this same pattern persisted in sequences obtained from amplifications using three different primer combinations, with sizes ranging from approximately 800 to 2700 bp. The sequence can be easily aligned and has a base composition similar to other published sequences for the Accipitridae. We could not find any evidence of pseudogene amplification, and since it has been suggested that PCR amplifications larger than 1.5 kilobases are likely to represent true mitochondrial amplifications [29], this site was coded as "M" (IUPAC code representing C and A) in all analyses, and it may represent an example of mitochondrial heteroplasmy.

### Phylogenetic analysis, single and combined datasets

None of the phylogenetic analyses supported the monophyly of the hawk genera *Leucopternis*, *Buteogallus *or *Buteo*. Phylogenies inferred from the subsets of mtDNA genes and combined data were largely congruent under all optimization criteria, and topologies differed mainly in resolution and nodal support. Although phylogenetic analysis of single gene subsets resulted in poorly resolved trees (data not shown), the nodes identified with high bootstrap support (> 75) or posterior probabilities (> 0.95) were entirely congruent with those identified in the combined analyses.

**Figure 1:**
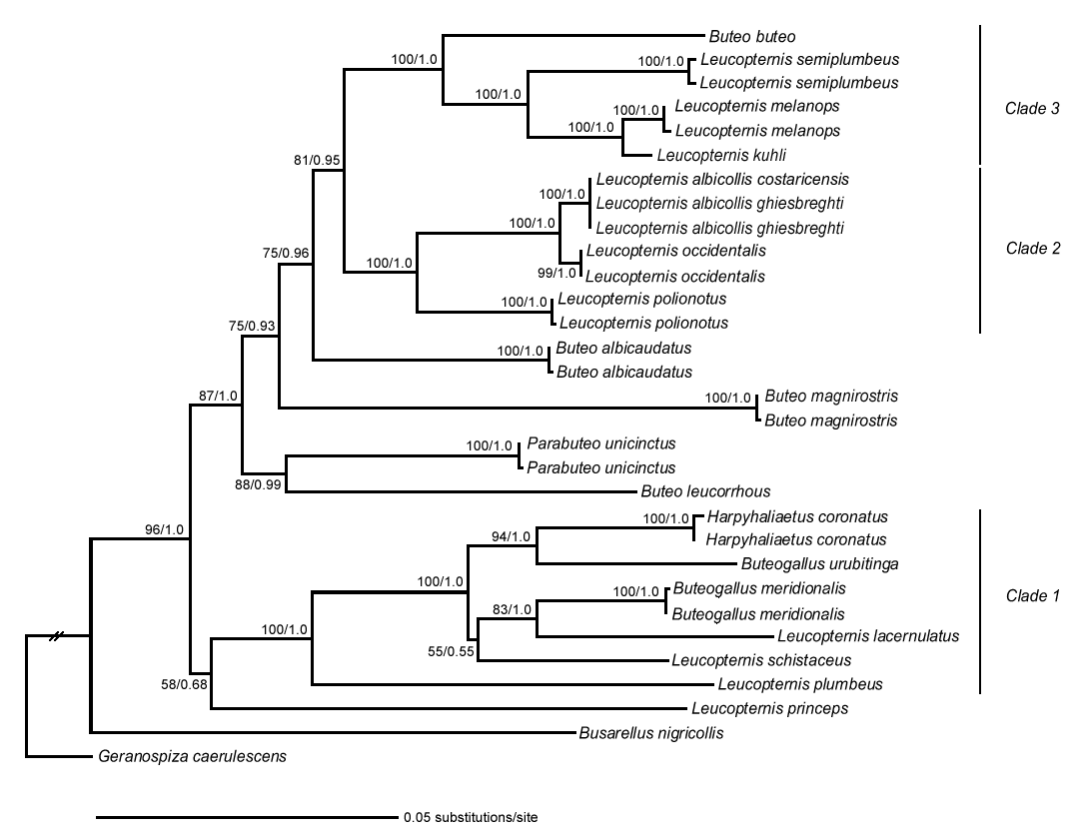
**Maximum likelihood topology of *Leucopternis *species and other Neotropical buteonines obtained from the combined data**. Numbers to the left of the node represent maximum likelihood bootstrap proportions (before slash) and Bayesian posterior probabilities (after slash). The branch leading to the outgroup was shortened for illustrative purposes.

The maximum likelihood (ML) inference of the total dataset resulted in one completely resolved tree with likelihood -ln 9813.2993 (figure 1), which was identical to the majority rule consensus topology of the Bayesian analysis (BA). Most nodes were highly supported by both bootstrap proportions and posterior probabilities. Maximum parsimony (MP) analyses of the total dataset resulted in two equally parsimonious trees (1531 steps, CI = 0.4899, RI= 0.6938) (trees not shown), which were also highly congruent with the ML and BA inferences. The main differences between the MP trees and the other combined analyses were the position of *L. princeps *basal to Clades 2 and 3 plus the remaining *Buteo *species and *Parabuteo unicinctus *(bootstrap support 68, data not shown), and the position of *L. schistaceus *basal to a poorly supported clade containing the sister pairs *Buteogallus meridionalis *and *L. lacernulatus*, and *H. coronatus *and *Buteogallus urubitinga *(bootstrap support < 50, data not shown).

Three major mtDNA clades containing *Leucopternis *species (figure 1) were recovered in all combined analysis, with high levels of statistical support in most cases. Clade 1 included *Buteogallus meridionalis, L. lacernulatus*, *Buteogallus urubitinga*, *H. coronatus*, *L. schistaceus and L. plumbeus*; *Leucopternis princeps *was basal to this Clade 1 in the ML and BA analyses, but with low support. The ML and BA analyses strongly supported a sister relationship between *L. lacernulatus *and *Buteogallus meridionalis*, and between *H. coronatus *and *B. urubitinga*; the latter sister pair was also highly supported by parsimony bootstrap (bootstrap support 98, data not shown). There was weaker support for a sister relationship between *L. schistaceus *and *Buteogallus meridionalis *plus *L. lacernulatus*, obtained in the ML and BA analyses. All phylogenetic trees inferred from the combined dataset establish an early split of *L. plumbeus *from the rest of the Clade 1.

Clade 2 comprised the two sampled *L. albicollis *subspecies (which carried identical mtDNA sequences), *L. occidentalis *and *L. polionotus*. Clade 3 was sister to Clade 2 and included *L. semiplumbeus*, *L. melanops *and *L. kuhli *sister to *Buteo buteo*. *Buteo albicaudatus*, *Buteo magnirostris*, *Parabuteo unicinctus *and *Buteo leucorrhous *were outside the sister relationship of Clade 2 and Clade 3 in all analyses. There was strong support in the ML, MP and BA trees obtained from the combined dataset for a sister relationship between *Buteo leucorrhous *and *P. unicinctus*.

**Figure 2:**
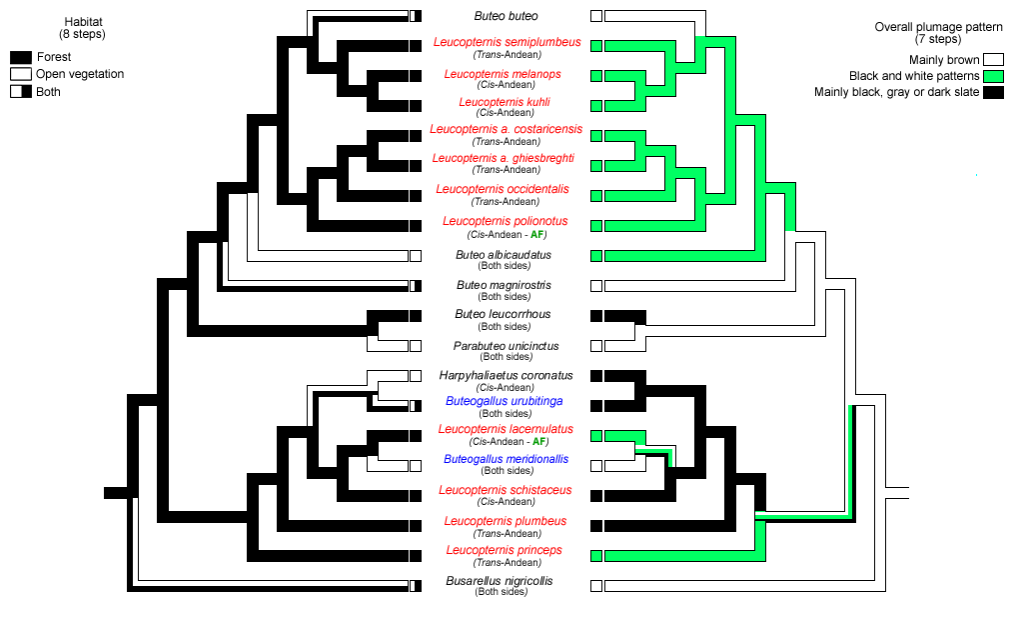
**Ancestral state reconstruction of habitat preferences and plumage characters of *Leucopternis *species and Neotropical buteonines. **Reconstructions determined by unordered parsimony using the ML topology obtained from the combined dataset. Species of *Leucopternis *and *Buteogallus *are indicated in red and blue. AF represents Atlantic Forest.

Shimodaira-Hasegawa tests were conducted on topologies constrained by the monophyly of all species in the genus *Leucopternis*, monophyly of species with the black and white plumage pattern, monophyly of all forest species, and reciprocal monophyly of the *cis- *and *trans*-Andean species. In all tests the constraint trees had a significantly poorer fit to the data than the unconstrained ML tree (P < 0.001). Ancestral state reconstructions of habitat and plumage traits onto the ML tree using unordered parsimony (Figure 2) showed that the black-and-white plumage characteristic to most *Leucopternis *species evolved at least twice in Neotropical buteonines, and shifts between forest and open habitats occurred at least four times.

## Discussion

### Phylogenetic relationships, novel groupings, and evolution of common plumage patterns

The mtDNA-based phylogenies for Neotropical buteonines reject the monophyly of the genera *Leucopternis*, *Buteogallus *and *Buteo*. Our analysis provides another example of the lack of correspondence between classic taxonomic groupings within the Accipitridae and molecular phylogenies [7,8,28]. The genus *Leucopternis *is a composite of three independent lineages, and several *Leucopternis *species are more closely related to other buteonine taxa than to each other.

One of the novel phylogenetic arrangements presented here is the sister relationship between *L. lacernulatu*s and *Buteogallus meridionalis*, rather than between *L. lacernulatus *and the sympatric Atlantic forest endemic *L. polionotus*, despite overall similarities in plumage pattern between the latter pair (see [30]). A proposed close relationship [1,4,12,30] between *L. lacernulatus *and the *L. melanops/L. kuhli *complex was not supported in any of our analyses.

Although close association of *L. lacernulatus *and *B. meridionalis *was unanticipated, the distinctiveness of the latter from other *Buteogallus *species has been recognized by its placement by some authors in the monotypic genus *Heterospizias *(e.g.,[10,11,30-34]). A close relationship between *Buteogallus urubitinga*, and *Harpyhaliaetus *eagles has been previously suggested [35,36], and the retention of the former in *Buteogallus *has been justified only by the bigger size of the *Harpyhaliaetus *species [35]. A recent higher-level molecular analysis of hawks and eagles [28] posited the paraphyly of the genus *Buteogallus*, and established a closer relationship of *Buteogallus urubitinga *and *Harpyhaliaetus *species than between the two *Buteogallus *species sampled (*B. urubitinga *and *B. anthracinus*). Given the polyphyletic relationships of *Buteogallus *as presented here it is clear that a complete re-assessment of the genus is required. It is worth noting that Amadon [4], Grossman and Hamlet [11], Brown and Amadon [12] and Ridgway [37] predicted the relatively close relationship of *L. schistaceus *and *L. plumbeus *to *Buteogallus *established in the mtDNA phylogeny presented here. However, we did not recover a sister relationship between the latter pair, sometimes considered subspecies [30] or members of a super-species complex (e.g., [9,38,39]).

The proximity of *L. polionotus*, *L. albicollis *and *L. occidentalis *is fully supported by our data, and has been strongly suggested in taxonomic revisions (e.g., [1,3,4,6,9,12,30,38,39]). Species limits in the *L. albicollis *complex have been a contentious taxonomic issue, and revision of this complex is needed. Similarly, a sister relationship between *L. melanops *and *L. kuhli *has been long suggested (e.g.,[1,4,6,30,38-40]), as well as the close relationship of both to *L. semiplumbeus *[12], based on similarities of plumage and external morphology. However, their close relationship to *Buteo buteo *is novel (see also [28]). In our study, *Buteo buteo *represents a genus containing several species of North American and Old World hawks (see [7]), and the nested position of this species within the Neotropical buteonines corroborates Amadon's [4] hypothesis of Neotropical origins for the entire buteonine lineage. The polyphyly of *Buteo *species in our study corroborates the results of Riesing *et al*. [7].

Our study includes all recognized *Leucopternis *species and represents the largest Neotropical buteonine taxonomic sample investigated to date. The polyphyly of *Leucopternis*, *Buteogallus *and *Buteo *indicates that current taxonomy does not reflect the phylogenetic history of the group. Only a complete buteonine phylogeny is likely to provide sufficient guidance regarding the nomenclatural issues concerning *Buteo*, *Leucopternis, Buteogallus *and *Harpyhaliaetus*, as well as fine scale biogeographic inferences. The predominantly black and white plumage pattern shared by several *Leucopternis *species (*L. polionotus, L. occidentalis, L. lacernulatus, L. melanops, L. kuhli, L. semiplumbeus, L. albicollis, L. princeps*) has evolved at least twice (figure 2), and the widespread occurrence of this pattern may result from plumage convergence in forested habitats. Similarly, *L. plumbeus *and *L. schistaceus *posses a slate plumage pattern very similar to that of *Rostrhamus *species, and all those species are associated with riparian habitats both within (*L. schistaceus, L. plumbeus, R. hamatus*) and outside forests (*R. sociabilis*). In order to test the hypothesis that the slate plumage results from selection in riparian habitats, additional analysis including *Rostrhamus *species is required. It has been shown that plumage characters under strong selection may evolve rapidly [41], and in some cases may represent evolutionary convergence instead of reflecting shared phylogenetic or phylogeographic history [42]. An overemphasis on the black and white plumage pattern influenced the grouping of *Leucopternis *species, and more generally our results indicate that plumage patterns alone may not be reliable taxonomic markers among Accipitridae species.

### Biogeography and habitat shifts

We can confidently reject the reciprocal monophyly of *cis- *and *trans-*Andean distributed buteonines (figure 2). At least three *cis-trans *(east-west) disjunctions were identified in all phylogenetic trees inferred from the combined dataset: (1) *L. semiplumbeus *(*trans-*) versus its Amazonian (*cis-*) sister clade, *L. melanops *plus *L. kuhli*, (2) *L. polionotus *(*cis-*) versus the two sampled subspecies of *L. albicollis *plus *L. occidentalis *(*trans-*), and (3) *L. plumbeus *(*trans-*) and the rest of Clade 1 (mostly *cis***-**Andean, but with *Buteogallus *species occurring on both sides). The uncertain position of *L. princeps *may hide a possible fourth disjunction. Three major hypothesis have been suggested to explain the differentiation of ancestral populations into *cis- *and *trans-*Andean lineages: the Andean Uplift Hypothesis [43], advocating separation of populations on either side of the mountains as a consequence of Andean orogeny; the Across Andes Dispersal Hypothesis [43-45] proposing long distance dispersal across the Andes as the cause of diversification, and the Forest Refugia Hypothesis, with historical expansions and contractions of forest corridors that linked forested lowlands on either side of the Andes [45] (see Brumfield and Capparella [46], Ribas *et al*. [47]). Lack of fossil calibrations for raptors, as well as rejection of clock-like evolution for the ATP8 and ATP6 dataset using a likelihood ratio test (data not shown) precluded calculation of divergence times for the hawk species analyzed here, and thus without a temporal framework we are unable to reject any of the Andean biogeography hypotheses based solely on mtDNA phylogenetic inference.

Few geographic barriers besides the Andes seem to define ranges of Neotropical buteonine species. The Amazon River has been suggested to delineate the distributions of *terra-firme *forest species [48,49], and may have played a role in the separation of *L. melanops *and *L. kuhli *north and south of the river, respectively (see [40]). However, there are recent and historical records of sympatry between *L. melanops *and *L. kuhli*, with observations of both species south of the Amazon River (Barlow *et al*. [50], Amaral *et al*. pers. obs.). Because of this fact it would appear that the Amazon River does not currently impose a strong barrier blocking *L. melanops *from occupying southern Amazon forests.

The putative contraction and expansion of lowland tropical forests associated with climate change has been promoted as one of the factors that explain avian areas of endemism (see [51] for limits and details on those areas), but most Neotropical buteonine species have geographic ranges that cross many of the proposed areas. Nonetheless, one might posit that the two Atlantic forest endemics, *L. lacernulatus *and *L. polionotus *represent species formed by the contraction and isolation of forest fragments during glacial episodes, but the mtDNA phylogenetic tree establishes that they are not sister species (Figure 1). The distant phylogenetic separation of these two *Leucopternis *species supports the proposal that the Atlantic Forest biota has complex origins [52].

Our mtDNA-based phylogenetic analysis of Neotropical buteonines (figure 2) also permits strong inference that forest and open-vegetation species are not reciprocally monophyletic. Furthermore, ecological shifts between forest and non-forest habitats occurred early and late in the Neotropical buteonine diversification. The phylogenetic tree presented in Figure 2 indicates that separation of the open-vegetation *Buteo albicaudatus *from *Leucopternis *forest species occurred early in the radiation compared to the more recent divergence of *Buteogallus meridionalis *from *L. lacernulatus *and *Parabuteo unicinctus *from *Buteo leucorrhous*. The geographic and altitudinal distributions of these more recently derived sister pairs overlap greatly, but the sister species occupy different habitats, either open-country or forest. This same pattern is reflected in the sister relationship between *Harpyhaliaetus coronatus *(savannah) and *Harpyhaliaetus solitarius *(forest, unavailable for this study) [28], although these species do not overlap much in their ranges.

Habitat shifts between sister species or groups of closely related species have been poorly explored in studies of avian historical biogeography in the Neotropics, mainly due to the predominance of forest lowland bird species in such studies (but see Garcia-Moreno and Cardoso da Silva [53] and Ribas *et al*. [54]). The pattern of habitat shifts between sister pairs can be consistent with both allopatric (Theory of Vanishing Refuges [55]) and parapatric models of speciation (Gradient Hypothesis [22]). Occurrence of parapatric speciation and divergence with gene flow has been a controversial issue (see Brown [56], Cracraft and Prum [57]), but explicit tests with vertebrates [58-61], including birds [62] have pointed to patterns consistent with between-habitat divergence in the presence of gene flow in tropical habitats. High mobility due to soaring and gliding flight, occurrence of several species in ecotones, and the pattern of sister relationships between forest and non-forest species suggest that buteonine species offer future opportunities to test alternative models of diversification in the Neotropics using phylogeographic data.

The determination of geographically structured areas of endemism in the Neotropics [51,63] has promoted allopatry as the principal mode of speciation in the Neotropics, with the Andean orogeny, rivers, and changes in forest cover serving as the principal vicariant events separating populations. Accumulation of phylogenetic and phylogeographic data for Neotropical birds suggests that the process of diversification is more complex [*e. g*. [42,64]], and that parapatric and sympatric models of speciation must be properly tested [19,23,58-62]. The family Accipitridae is extensively represented in most Neotropical habitats, and offers opportunities to explore the radiation of an ecologically diverse group with high dispersal capabilities. Further phylogenetic and phylogeographic studies of diurnal raptors, as well as other groups representing varying degrees of vagility and occurrence in habitats other than forests, will permit more explicit tests of the role of alternative modes of speciation acting on Neotropical birds, and refinement of general explanations for the origin and maintenance of Neotropical biodiversity.

## Conclusion

Our mtDNA-based inference of Neotropical buteonine phylogeny establishes a polyphyletic relationship among the hawk genera *Leucopternis*, *Buteogallus *and *Buteo*. Thus the phylogeny indicates that the current taxonomy of the Accipitridae is not a good guide to the evolutionary relationships of species in the group, and identifies a need for further systematic analysis of the family at all taxonomic levels. We do not propose nomenclatural modifications, since only a complete buteonine analysis would permit such taxonomic changes. Nonetheless, our results coupled to earlier work predict some of the nomenclatural changes that will undoubtedly be forthcoming, and also establish that plumage has been overemphasized in defining the taxonomy of the Accipitridae. Finally, we conclude that shifts between forest and non-forest habitats, as well as movement across the Andes, have occurred more than once during the Neotropical buteonine diversification.

## Methods

### Taxon sampling, DNA extraction, amplification and sequencing

We sampled a total of 31 specimens, comprising all 10 recognized species of the genus *Leucopternis *(*L. lacernulatus*, *L. polionotus*, *L. semiplumbeus, L. plumbeus, L. occidentalis, L. schistaceus, L. princeps, L. melanops, L. kuhli, L. albicollis)*, and including two sub-species of *L. albicollis *(*L. a. costaricensis *and *L. a. ghiesbreghti*), as well as almost all Neotropical buteonine genera (representing 15 of the 21 species of the Neotropical "sub-buteonines" *sensu *Amadon [4]), plus four *Buteo *species. We chose *Geranospiza caerulescens *as an outgroup based on a recent higher-level analysis of Accipitridae [28]. When possible, we included two individuals per taxon. Nomenclature follows the South American Classification Committee of the American Ornithologists' Union [65]. Sequences of *Buteo buteo *were obtained from Genbank [NC_003128]. Tissue, feather and blood samples were obtained from specimens collected in the field, museum tissue collections, and captive birds (see Additional file 1: Table 1). Known localities of origin, feathers and photographs are available for most captive specimens.

DNA extraction, amplification and sequencing were performed at the Universidade de São Paulo (Brazil), the Smithsonian Tropical Institute (Panama) and the Royal Ontario Museum (Canada) based on earlier protocols [66]. DNA extraction followed Bruford *et al*. [67], or via the DNeasy kit (Qiagen); for feather samples, we added 30 ug of dithiothreitol to the digestion buffer. We sequenced four mitochondrial genes: a portion of the 12S ribosomal RNA gene (12S, longest sequence of 833 bp), the complete ATP synthase F0 subunit 8 (ATP8, 168 bp) and subunit 6 (ATP6, 684 bp) genes, as well as the complete NADH dehydrogenase subunit 6 (ND6, 519 bp) using several primer pair combinations via polymerase chain reaction (PCR) (table 2). In few cases, weak amplification products were re-amplified using internal primers. Both strands of the amplified products were sequenced.

**Table 2:** Primers used in the study.

**Target region**	**Primer name**	**Sequence (5'to 3')**	**Reference**
**12S**	LPHE1248	AAAGCATGGCACTGAAGAYGCCAAG	E. Tavares, unpublished
	12SL1735	GGATTAGATACCCCACTATGC	Miyaki *et al*. [75]
	12SHC	CCGCCAAGTCCTTAGAGTTT	Eberhard *et al*. [76]
	12SH2181	GGCTTGTGAGGAGGGTGACGGGC	C. Ribas, unpublished
	H2294VAL	CTTTCAGGTGTAAGCTGARTGC	J. Patane, modified from Sorenson *et al*. [77]
	16S2-	ATCCCTGGGGTAGCTTGGTCC	Haring *et al*. [78]
	16SH3309	TGCGCTACCTTCGCACGGT	Miyaki *et al*. [75]
	H4017	GCTAGGGAGAGGATTTGAACCTC	Sorenson *et al*. [77]
**ATP8/6**	CO2GQL	GGACAATGCTCAGAAATCTGCGG	Eberhard and Bermingham [79]
	TLYS9051	CACCAGCACTAGCCTTTTAAG	Fleischer *et al*. [80]
	A6PWL	CCTGAACCTGACCATGAAC	Eberhard and Bermingham [79]
	CO3HMH	CATGGGCTGGGGTCRACTATGTG	Eberhard and Bermingham [79]
	ARG11145	TTTGTTGAGCCGAAATCAACTGTCT	Present study
**ND6**	TPROFWD	ATCACCAACTCCCAAAGCTGG	Riesing *et al*. [7]
	TGLUREV	AAGTTTACAACGGCGATTTTTC	Riesing *et al*. [7]
	YCR2REV	GGTTACATGGTTTGGTAGGGG	Riesing *et al*. [7]

### Alignment and phylogenetic analysis

Multiple strands obtained for each specimen were assembled in CodonCode Aligner v. 1.3.4 (CodonCode Corporation) or Sequencher v. 4 (Gene Codes Corporation). Contigs were exported and alignment performed in Clustal X 1.83 [68] with default parameters. The 12S alignment had 21 indels, which consisted mostly of autapomorphies and sites of ambiguous alignment; these were removed from all analyses. All single marker and combined datasets were tested for significant departures from average base frequencies with PAUP* 4b10 [69], using only variable sites. Uncorrected codon-based (ATP8, ATP6, ND6) and total (12S) transition and transversion distances were plotted against Kimura-2-parameters distances using the software Dambe v4.2 [70] to evaluate the effect of multiple substitutions in each dataset. We implemented a partition homogeneity test in PAUP*, using only variable sites with 1000 replicates with random additions, to evaluate the congruence of the phylogenetic signal between the different genes. Because the latter test did not detect significantly-different phylogenetic signal among the partitions (P = 0.29), all four genes were combined into a single combined dataset. Separated analyses of subsets of single gene region were also performed to evaluate the concordance among those datasets. The ATP8 and ATP6 genes overlap by 10 bp; however in all phylogenetic analyses of the combined dataset and the single gene region of ATP (subunit 8 plus subunit 6) this region was considered only once. We performed phylogenetic reconstructions using maximum likelihood (ML) and maximum parsimony (MP) implemented in PAUP*, and Bayesian analysis (BA) with MrBayes v3.1.1 [71], to evaluate the concordance of topologies obtained under different optimization criteria. ML and MP heuristic searches were performed using 1000 and 10 random additions of sequences, respectively. Nonparametric bootstrapping was performed to assess branch support (100 replicates with single random additions for the ML analysis and 1,000 replicates with 10 random additions for the MP analysis). Modeltest v3.7 [72] was used to choose among evolutionary models of DNA substitution for ML and BA analyses using a hierarchical likelihood ratio test. Modeltest determined that the TrN+I+G model was the best fit for the total dataset with base frequencies of A = 0.3217, C = 0.3521, G = 0.1235, T = 0.2027, a gamma shape parameter of 1.0617 and proportion of invariable sites of 0.5631. The BA analyses of the combined dataset was run with individual likelihood for each of the three gene regions (12S, ATP 6 and ATP 8, and ND6) as selected by Modeltest (TrN+I+G, HKY+I+G, TrN+G), which were the same models used for subset ML and BA analyses. MrBayes was run with four chains for 4,000,000 generations with trees sampled every 100 generations, replicated four times. All runs reached stationarity around 400 sampled generations, so we discarded the first 40000 generations as a "burnin"; a consensus topology was created with all the remaining sampled generations.

To determine whether our data support monophyly of *Leucopternis*, monophyly of forest species, monophyly of species presenting black and white plumage patterns, or reciprocal monophyly of *trans- *and *cis-*Andean species, we compared alternative constraint topologies to the ML tree using the nonparametric Shimodaira-Hasegawa test [73] implemented in PAUP*. Ancestral states of habitat and general plumage pattern (according to Thiollay [1], Fergusson-Lees and Christie [6] and Sibley and Monroe [39]) were mapped onto the ML tree inferred from the combined dataset using unordered parsimony in Mesquite v1.05 [74].

## Authors' contributions

FSRA conceived the study, carried out most of the data collection and phylogenetic analysis, and drafted the manuscript. MJM carried out the data collection from the LSUMZ samples, and made substantial contributions to the manuscript. LFS obtained part of the samples, and made substantial contributions to the manuscript. EB made substantial contributions to the manuscript. AW helped to conceive the study, participated in its design and coordination and helped to draft the manuscript. All authors read and approved the final manuscript.

## Supplementary Material

Additional File 1 **Table 1. Samples used in the study. **The classification follows Remsen *et al*. [65]. Abbreviations: LGEMA = Laboratório de Genética e Evolução Molecular de Aves, Universidade de São Paulo; LSUMZ = Louisiana State University, Museum of Natural Science; ANSP = Academy of Natural Sciences of Philadelphia; MPEG = Museu Paraense Emílio Goeldi; MZUSP = Museu de Zoologia da Universidade de São Paulo; IBUSP = Instituto de Biociências, Universidade de São Paulo. Click here for file
