# Supplementary material for: Polyphyly of the hawk genera Leucopternis and Buteogallus (Aves, Accipitridae): multiple habitat shifts during the Neotropical buteonine diversification
Source: BMC Evol Biol. 2006 Feb 7;6:10. doi: 10.1186/1471-2148-6-10 (PMC1413559; doi:10.1186/1471-2148-6-10)
Supplement: Additional File 1 — Table 1. Samples used in the study. The classification follows Remsen et al. [65]. Abbreviations: LGEMA = Laboratório de Genética e Evolução Molecular de Aves, Universidade de São Paulo; LSUMZ = Louisiana State University, Museum of Natural Science; ANSP = Academy of Natural Sciences of Philadelphia; MPEG = Museu Paraense Emílio Goeldi; MZUSP = Museu de Zoologia da Universidade de São Paulo; IBUSP = Instituto de Biociências, Universidade de São Paulo. [file 1471-2148-6-10-S1.rtf]

Species

	Sample ID	Locality	Voucher and Origin	DNA Source	GenBank Acess. Numb.	
					12S	ATP8/6	ND6	
Busarellus nigricollis	LGEMA F119	Caracaraí, Roraima, Brazil	MPEG 56252	Tissue	[DQ148338]	[DQ148368]	[DQ148308]	
Buteo albicaudatus	LGEMA F46	Joima, Minas Gerais, Brazil	SOS Falconiformes  (captivity)	Feathers	[DQ148343]	[DQ148373]	[DQ148313]	
Buteo albicaudatus	LGEMA F85	E.E. Uruçuí-Una, Piauí, Brazil	MZUSP 75202	Tissue	[DQ148354]	[DQ148384]	[DQ148324]	
Buteo leucorrhous	LGEMA F103	Florianópolis, Santa Catarina	S.O.S. Falconiformes (captivity)	Feathers	[DQ148337]	[DQ148367]	[DQ148307]	
Buteo magnirostris	LGEMA F38	Wenceslau Brás, Paraná	Voucher  Pending  (IBUSP)	Tissue	[DQ148339]	[DQ148369]	[DQ148309]	
Buteo magnirostris	LGEMA F74	São Paulo, São Paulo, Brazil	Voucher  Pending (IBUSP)	Tissue	[DQ148352]	[DQ148382]	[DQ148322]	
Buteogallus meridionalis	LGEMA F49	Unknown	Zoológico de Paulínia  (captivity)	Blood	[DQ148344]	[DQ148374]	[DQ148314]	
Buteogallus meridionalis	LGEMA F54	São Paulo, São Paulo, Brazil	Voucher  Pending (IBUSP)	Tissue	[DQ148347]	[DQ148377]	[DQ148317]	
Buteogallus urubitinga	LGEMA F72	Unknown	Zoológico de São Paulo (captivity)	Blood	[DQ148351]	[DQ148381]	[DQ148321]	
Geranospiza caerulescens	LGEMA F84	Uruçuí-Una, Piauí, Brazil	MZUSP 75203	Tissue	[DQ148353]	[DQ148383]	[DQ148323]	
Harpyhaliaetus coronatus	LGEMA F44	Minas Gerais, Brazil	S.O.S. Falconiformes  (captivity)	Blood	[DQ148341]	[DQ148371]	[DQ148311]	
Harpyhaliaetus coronatus	LGEMA F45	Ubá, Minas Gerais, Brazil	S.O.S. Falconiformes  (captivity)	Feathers	[DQ148342]	[DQ148372]	[DQ148312]	
Leucopternis albicollis costaricensis	LGEMA F52	Darien, Panama	Peregrine Fund (captivity)	Feathers	[DQ148345]	[DQ148375]	[DQ148315]	
Leucopternis albicollis ghiesbreghti	LGEMA F56	Veracruz, Mexico	Pronatura Veracruz (captivity)	Blood	[DQ148349]	[DQ148379]	[DQ148319]	
Leucopternis albicollis ghiesbreghti	LGEMA F57	Mexico	ZOOMAT (captivity)	Blood	[DQ148350]	[DQ148380]	[DQ148320]	
Leucopternis kuhli	LSUMZ B-4598	Loreto Department, Peru	LSUMZ B-4598	Tissue	[DQ148360]	[DQ306261]	[DQ148330]	
Leucopternis lacernulatus	LGEMA F39	Praia Grande, São Paulo, Brazil	MZUSP 76193	Tissue	[DQ148340]	[DQ148370]	[DQ148310]	
Leucopternis melanops	ANSP 7554	Guyana, Iwokrama Reserve, Iwokrama mountains	ANSP 7554	Tissue	[DQ148335]	[DQ148365]	[DQ148305]	
Leucopternis melanops	ANSP 7883	Guyana, Iwokrama Reserve, Kabocalli Landing	ANSP 7883	Tissue	[DQ148336]	[DQ148366]	[DQ148306]	
Leucopternis occidentalis	ANSP 2901	Ecuador, South of Ayamee	ANSP 2901	Tissue	[DQ148333]	[DQ148363]	[DQ148303]	
Leucopternis occidentalis	ANSP 4321	Ecuador, 30km south of Chontaduro, west bank of Rio Verde.	ANSP 4321	Tissue	[DQ148334]	[DQ148364]	[DQ148304]	
Leucopternis plumbeus	ANSP 2241	20 km NNW of Alto Tambo
Ecuador	ANSP 2241	Tissue	[DQ148332]	[DQ148362]	[DQ148302]	
Leucopternis polionotus	LGEMA F53	Unknown	Zoológico de Curitiba   (captivity)	Feathers	[DQ148346]	[DQ148376]	[DQ148316]	
Leucopternis polionotus	LGEMA F55	Unknown	Zoológico de Curitiba   (captivity)	Feathers	[DQ148348]	[DQ148378]	[DQ148318]	
Leucopternis princeps	LSUMZ B-11751	Esmeraldas Province, Ecuador	LSUMZ B-11751	Tissue	[DQ148357]	[DQ148387]	[DQ148327]	
Leucopternis schistaceus	LSUMZ B-4946	Loreto Department, Peru	LSUMZ B-4946	Tissue	[DQ148361]	[DQ148390]	[DQ148331]	
Leucopternis semiplumbeus	LSUMZ B-2291	Darién Province, Panama	LSUMZ B-2291	Tissue	[DQ148358]	[DQ148388]	[DQ148328]	
Leucopternis semiplumbeus	LSUMZ B-2326	Darién Province, Panama	LSUMZ B-2326	Tissue	[DQ148359]	[DQ148389]	[DQ148329]	
Parabuteo unicinctus	LGEMA F91	Baixada Santista, São Paulo, Brazil	Orquidário de Santos (captivity)	Feathers	[DQ148355]	[DQ148385]	[DQ148325]	
Parabuteo unicinctus	LGEMA F93	Ilha do Governador, Rio de Janeiro, Brazil	ABFPAR (captivity)	Feathers	[DQ148356]	[DQ148386]	[DQ148326]	
Additonal file 1  -  Samples used in the study. 
The classification follows Remsen et al. [65] Abbreviations: LGEMA = Laboratório de Genética e Evolução Molecular de Aves, Universidade de São Paulo; LSUMZ = Louisiana State University, Museum of Natural Science; ANSP = Academy of Natural Sciences of Philadelphia; MPEG = Museu Paraense Emílio Goeldi; MZUSP = Museu de Zoologia da Universidade de São Paulo; IBUSP = Instituto de Biociências, Universidade de São Paulo.
